# The role of OCT-A in retinal disease management

**DOI:** 10.1007/s00417-018-4109-3

**Published:** 2018-09-03

**Authors:** Francisco J. Rodríguez, Giovanni Staurenghi, Richard Gale, On behalf of the Vision Academy Steering Committee, Bora Eldem, Alex Hunyor, Antonia Joussen, Adrian Koh, Jean-François Korobelnik, Paolo Lanzetta, Anat Loewenstein, Monica Lövestam-Adrian, Rafael Navarro, Annabelle A. Okada, Ian Pearce, Giovanni Staurenghi, Francisco J. Rodríguez, Sebastian Wolf, David T. Wong

**Affiliations:** 1 Fundación Oftalmológica Nacional, Calle 50, #13–50, Bogotá, Colombia; 2 Department of Ophthalmology, University of Rosario School of Medicine, Bogotá, Colombia; 3 University Eye Clinic, Department of Biomedical and Clinical Sciences ‘Luigi Sacco’, University of Milan, Milan, Italy; 4 Department of Ophthalmology, York Teaching Hospital NHS Foundation Trust, York, UK; 5 Department of Health Sciences, University of York, York, UK

**Keywords:** Optical coherence tomography angiography, OCT-A, Non-invasive, Retinal diseases, Imaging modality

## Abstract

Optical coherence tomography angiography (OCT-A) is a non-invasive, non-dye-based imaging modality that has the potential to enhance our understanding of retinal diseases. While this rapidly advancing imaging modality offers great potential, there is a need for community-wide understanding of the range of technologies and methods for interpreting the images, as well as a need to enhance understanding of images from disease-free eyes for reference when screening for retinal diseases. Importantly, clinical trials have been designed without OCT-A-based endpoints; therefore, caution is required when making treatment decisions based on OCT-A imaging alone. With this in mind, a full understanding of the advantages and limitations of OCT-A will be vital for effective development of the technique within the field of ophthalmology. On behalf of the Vision Academy Steering Committee (sponsored by Bayer), this publication summarizes the views of the authors on the current use of OCT-A imaging and explores its potential for future applications in research and clinical practice.

## Introduction

Optical coherence tomography (OCT) has progressed rapidly since its implementation in the field of ophthalmology. It is becoming a widely used imaging tool in the diagnosis of choroidal neovascularization (CNV) or macular edema in different retinal and choroidal diseases. Using the same technology, OCT angiography (OCT-A) was introduced as a means for visualizing retinal and choroidal vasculature. Unlike fluorescein angiography (FA) and indocyanine green angiography (ICGA), which use intravenous dyes to fill the fundus vasculature and permit its visualisation, OCT-A utilizes motion-contrast imaging, calculating differences in backscattered OCT signal intensity between sequential scans of the same area [1–3]. This non-invasive ocular imaging technique can resolve the distances of reflective structures within tissues, producing high-resolution, cross-sectional scans of vascular flow in seconds [2–4]. However, there is, as yet, little consensus within the retinal specialist community on the appropriate use of OCT-A for diagnosis or monitoring, and current treatment recommendations state that the use of OCT-A is not essential for good patient management.

As an emerging technology that could potentially have a significant impact on the clinical management of retinal disease, the technique of OCT-A falls within the scope of the Vision Academy. The Vision Academy is an initiative that brings together global leaders in ophthalmology, providing a forum to share existing skills and knowledge. With defined activity programs, the Vision Academy seeks to address key unmet needs in the field of retinal disease, providing outputs to build best practice and lead the wider community in the drive towards optimized, compassionate patient care. Members of the Vision Academy Steering Committee (see “Acknowledgments” section) met in 2016 to discuss the use of OCT-A for the management of retinal disease, with particular focus on its current utility, limitations, and potential for future application in research and clinical practice. In this review, we summarize the views of the Vision Academy Steering Committee, focusing on the use of OCT-A in neovascular age-related macular degeneration (nAMD).

## Understanding the challenges of OCT-A

OCT-A imaging provides a detailed view of retinal vasculature for qualitative and quantitative analysis of retinal structure and microvascular function [2]. Unlike FA and ICGA, OCT-A has the ability to easily segment different layers of the retina, making it an exciting tool for developing our understanding of retinal diseases [2]. However, without official guidance for OCT-A use in retinal disease management, there remains a lack of clarity on the appropriate interpretation of disease characteristics, as well as a lack of understanding of ‘normal’ OCT-A images of disease-free eyes for use as a reference point when making diagnoses and treatment decisions.

**Fig. 1:**
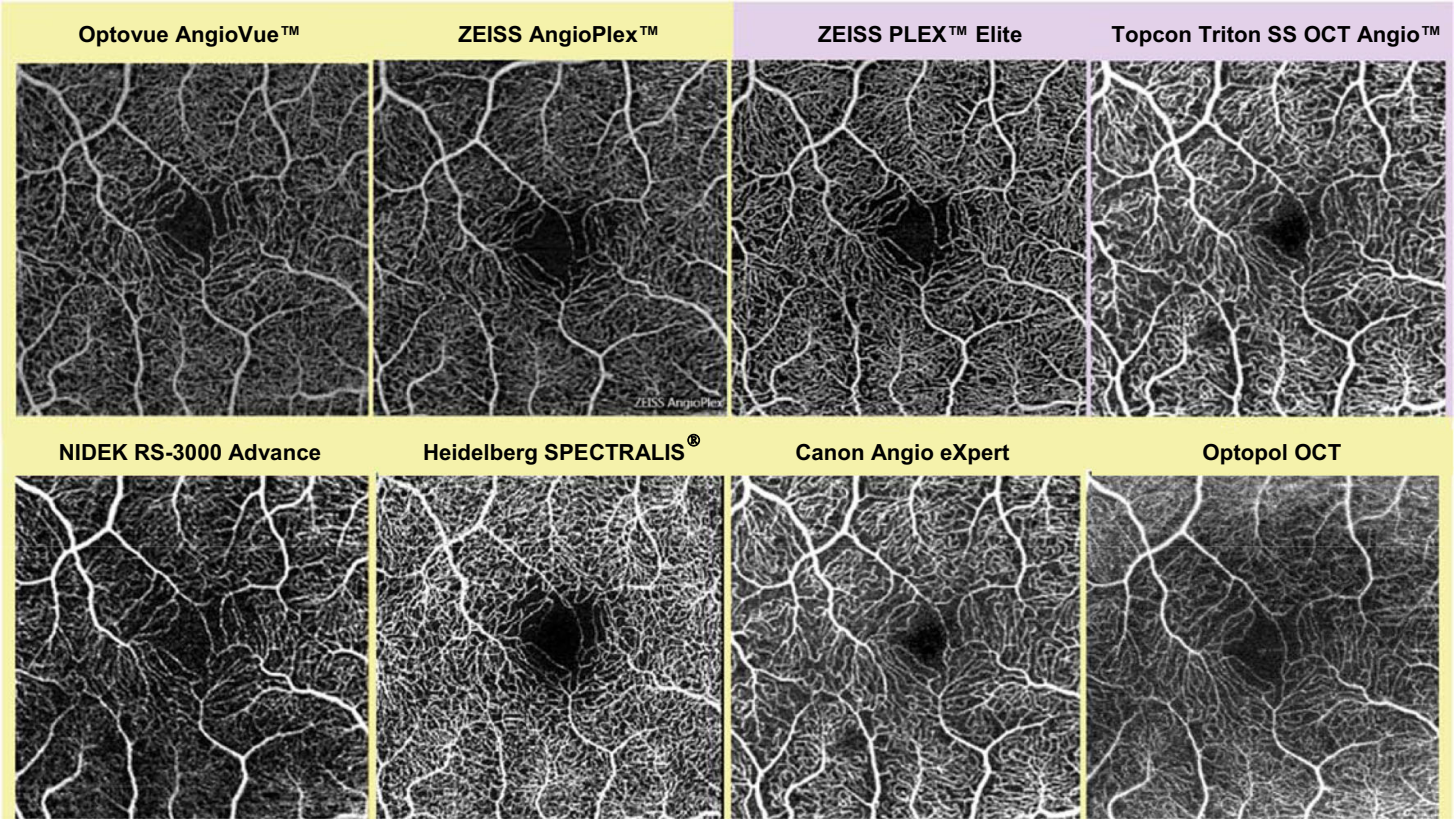
Images of normal retinal microvasculature taken using a range of available OCT-A technologies. *OCT* optical coherence tomography, *OCT-A* optical coherence tomography angiography, *SS OCT* swept-source optical coherence tomography. Source: Images provided by Giovanni Staurenghi

Despite only emerging in recent years, OCT-A is already widely used in clinical practice and is increasingly used as a diagnostic tool for retinal disease; for example, OCT-A can be used to identify the presence of CNV in nAMD and to identify areas of non-perfusion associated with diabetic retinopathy [2]. However, the technology is advancing at a much greater rate than the retinal specialist community’s experience of the technique, an issue that is confounded by the wide range of OCT-A technologies available. Currently, there are a number of available methods for OCT-A image acquisition, including amplitude-decorrelation and phase-variance algorithms (Table 1), as well as two methods for OCT-A image averaging [4–8, 12]. Figure 1 shows images of the microvasculature of a healthy retina in one patient, taken using a range of OCT-A technologies available on the market.

**Table 1:** A summary of available algorithms for OCT-A imaging

OCT-A algorithm	Definition	Developer (OCT device)
OMAG [5–7]	Optical microangiography	ZEISS (AngioPlex™)/University of Washington
CODAA [8]	Complex OCT signal differential analysis angiography	NIDEK (AngioScan)
SSADA [2, 4–7, 9]	Split-spectrum amplitude-decorrelation angiography	Optovue (AngioVue™)
FSADA [6]	Full-spectrum amplitude-decorrelation angiography	Canon (Angio eXpert)
SPECTRALIS^®^ OCT-A [6]	Full-spectrum probabilistic approach	Heidelberg Engineering (SPECTRALIS^®^)
OCTARA [6]	OCT angiography ratio analysis	Topcon (Triton SS OCT Angio™)
PRD-OCT [10]	Phase-resolved Doppler OCT	University of Amsterdam
PV-OCT [6]	Phase-variance OCT	California Institute of Technology
UHS SS OCT [11]	Ultra-high-speed swept-source OCT with variable interscan time analysis (VISTA)	Massachusetts Institute of Technology

*OCT* optical coherence tomography, *OCT-A* optical coherence tomography angiography

While OCT-A is not considered essential for good patient management, retinal centers will inevitably feel under pressure to follow the OCT-A trend. In recent years, there has been an exponential increase in the number of publications on OCT-A in the context of retinal disease, reflecting the interest of the clinical community in this technology.

OCT-A in combination with structural OCT has been shown to be more effective than either FA or OCT-A alone for the evaluation of macular complications associated with retinal disease [13]. However, there is a growing concern that physicians may be making treatment decisions based on OCT-A alone, which could have a detrimental effect on patient care. While the acquisition of OCT-A scans is fast, image interpretation can be time-consuming, with the need to analyze data from all available B-scans and the entire retinal slab to reliably avoid misinterpretation of image artifacts [1]. Furthermore, it remains unclear whether OCT-A is useful in all cases, or if it is better suited to particular subgroups of patients with retinal disease and specific pathologies. This situation is further complicated by the number of OCT-A technologies available; each has its own advantages and limitations when imaging different retinal disease pathologies, yet there is no clear consensus on which patient subgroups they are most suited to.

Rather than attempting to identify a superior technology from the OCT-A platforms available, our focus should instead be on optimizing the use of existing equipment and accurately interpreting the resulting scans. Currently, there are no standardized protocols for image acquisition, although some physicians are employing their own protocols, using the specific OCT-A platform in their center. With little shared community-wide experience of image interpretation, a lack of defined protocols may lead to inconsistencies in clinical practice.

**Fig. 2:**
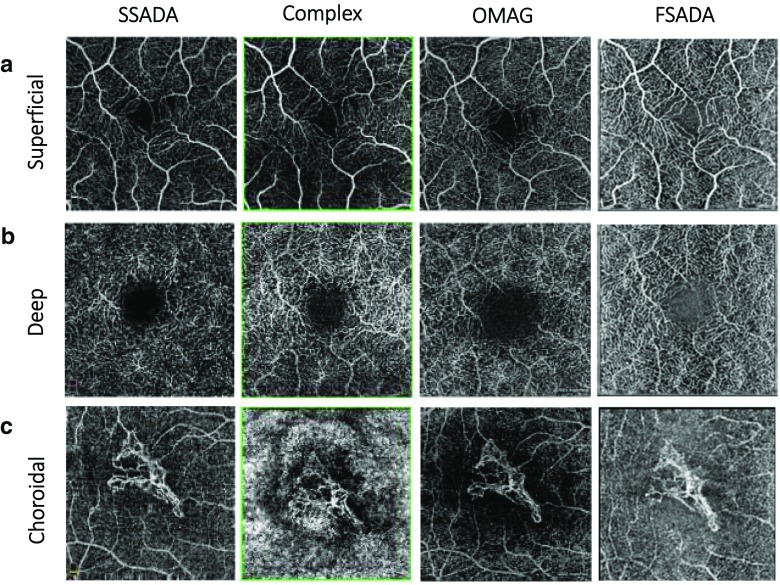
Microvasculature resolution in the superficial and deep retinal vessels of normal subjects (**a** and **b**, respectively), and the choroidal layer of a patient with CNV (**c**), visualized using a range of available OCT-A algorithms. *CNV* choroidal neovascularization, *FSADA* full-spectrum amplitude-decorrelation angiography, *OMAG* optical microangiography, *SSADA* split-spectrum amplitude-decorrelation angiography. Source: Presented by Giovanni Staurenghi at the Vision Academy Annual Meeting 2016; Barcelona, Spain, March 12–13, 2016

A major benefit for OCT-A is its ability to scan the deep tissue layers of the eye, notably the choroid [1]. However, it is often challenging to quantify retinal morphology in patients with nAMD presenting with fibrovascular pigment epithelial detachment (PED) or mixed fibrovascular PED, or abnormalities of choroidal microvasculature in cases of CNV. Figure 2 demonstrates the visual capabilities of a range of different OCT-A algorithms utilized in clinical practice to resolve structures across multiple tissue levels, including their ability to visualize deep regions of neovascularization in the choroid (*bottom row*). Upgrades in software for image processing and visualization are continuously being produced and are improving the quality of OCT-A images. However, variation in image quality across different OCT-A algorithms further highlights the need for standardized protocols for imaging the different retinal layers in each disease indication.

## OCT-A as a complementary diagnostic tool

The effectiveness of OCT-A has been demonstrated in principle for the diagnosis of CNV in patients with nAMD [14]. OCT-A is an additional technique in the current portfolio of imaging modalities, with potential for use alongside FA, ICGA, and conventional OCT as part of a multimodal diagnostic process for optimal evaluation of disease prognosis.

**Fig. 3:**
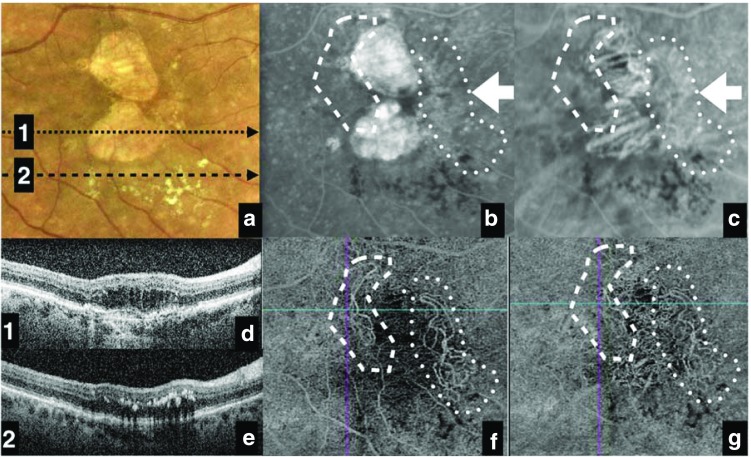
A case study of a patient presenting with visual loss in the right eye, demonstrating a change in overall diagnosis after implementation of OCT-A as part of a multimodal imaging approach. Retinal imaging was provided by **a** color fundus photography, **b** FA, **c** ICGA, and **d**, **e** OCT, and led to an initial diagnosis of suspicious CNV (regions marked with dotted and dashed lines in images **b** and **c**). Further imaging by **f**, **g** OCT-A led to a final diagnosis of two different CNVs: one active (dotted line) and one inactive (dashed line) in AMD. In image **a**, arrows 1 and 2 represent the locations of cross-sectional B-scans (images **d** and **e**, respectively). *AMD* age-related macular degeneration, *CNV* choroidal neovascularization, *FA* fluorescein angiography, *ICGA* indocyanine green angiography, *OCT* optical coherence tomography, *OCT-A* optical coherence tomography angiography. Source: Images provided by Giovanni Staurenghi

While current treatment guidelines state that OCT-A is not essential for effective patient management, it could prove a useful tool for physicians who currently make retreatment decisions without the use of an angiogram. For physicians who already use angiography to inform their treatment decisions, OCT-A should be considered a useful complementary technique that can provide additional information for diagnosis. OCT-A imaging of subjects who were previously diagnosed using more traditional methods (such as FA or conventional OCT) may ultimately lead to a change in diagnostic algorithms based on the additional information available from the resulting images (see Fig. 3).

## Current limitations of OCT-A imaging in retinal disease

Several practical limitations of OCT-A require further consideration.

In a study of healthy subjects, Matsunaga et al. demonstrated that 3 × 3 mm OCT-A images are at least equivalent in quality to scans obtained using FA [15]. However, a small scan size, with a limited field of view, may limit the use of OCT-A for disease screening. While the standard imaging area of the retinal surface with OCT-A is 3 × 3 mm, some physicians are opting to acquire larger scan sizes, e.g. 6 × 6 mm or 8 × 8 mm [2]. However, a widened field of view may decrease the image quality because some software utilizes the same number of B-scans across all scanning areas [2]. In order to achieve a field of view of the same size as these traditional imaging techniques while retaining a high level of resolution, physicians may have to stitch together multiple images, using montage OCT-A, to visualize areas of the retinal vasculature [16, 17].

OCT-A imaging can only detect blood flow above a minimum threshold, since it relies on differences between consecutive OCT B-scans to determine flow. With current OCT-A technologies, increasing the time interval between consecutive B-scans would greatly increase the risk of artifacts caused by eye motion, resulting in a limited ability to detect slowly flowing structures (which are often seen in cases of microaneurysm or fibrotic CNV) [2]. Instead, in some cases where a slowly flowing lesion is not visualized using the first and second OCT B-scans, the high-speed scanning capabilities of OCT-A systems that utilize variable interscan time analysis (VISTA) allow an image to be processed using the first and third B-scans, thereby decreasing the minimum threshold without increasing the risk of motion artifacts [2].

Similarly, restricted visualization of slow-filling polyps may limit the reliability of OCT-A for use as a primary diagnostic tool in patients with polypoidal choroidal vasculopathy; however, a study by Inoue et al. demonstrated the potential for combining *en face* and cross-sectional OCT-A to resolve polypoidal structures [18].

Another limitation of OCT-A may be its inability to accurately determine the extent of vascular leakage. This could limit the use of OCT-A alone in the identification of changes in vascular permeability associated with diseases such as nAMD, diabetic macular edema, and retinal vein occlusion.

## OCT-A imaging artifacts

**Fig. 4:**
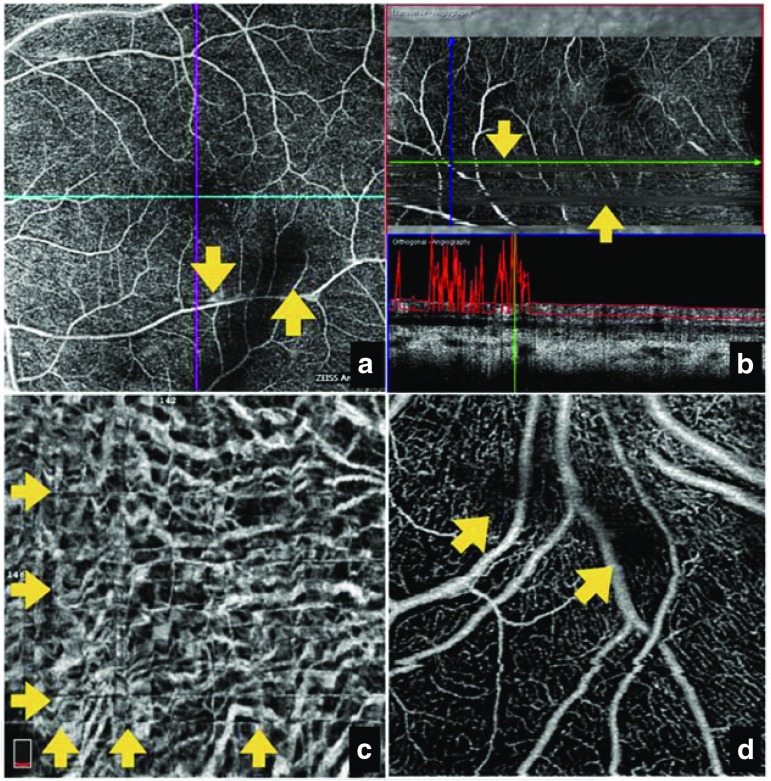
OCT-A imaging distortions caused by **a** ocular motion, revealing loss of detail despite the high signal score, with apparent doubling of vessels; **b** segmentation failure, leading to visualization of vessels from different layers in one image that does not reflect actual anatomy; **c** criss-cross image defects introduced after software-based motion correlation to compensate for ocular motion artifacts; **d** intrinsic properties of the eye, such as vitreous ‘floaters’, causing a loss of signal with media opacity. Yellow arrows indicate regions of interest. *OCT-A* optical coherence tomography angiography. Source: Images provided by Giovanni Staurenghi

An understanding of OCT-A image artifacts is essential for accurate assessment of retinal disease pathologies. There are a number of known causes of OCT-A image artifacts, including distortions caused by errors in image processing and display, or ocular motion (Fig. 4) [19]. Certain characteristics of the eye may also cause artifacts; for example, given the high prevalence of myopia among patients, eyes may have a high refractive error and OCT-A light beams may be non-perpendicular to the retinal surface [19].

Figure 4a demonstrates image distortion caused by ocular motion, revealing doubling of retinal vasculature and a subsequent inability to produce a valid assessment of disease pathology. While this remains a limitation of OCT-A, software-based motion correction methods have been developed to manage image distortions caused by eye movement; however, these can introduce criss-cross image defects (Fig. 4c) [19, 20].

Prominent media and vitreous opacities (‘floaters’), as well as superficial retinal vessels, can cause shadow artifacts, making it difficult to clearly visualize the deeper retinal layers (Fig. 4d) [9, 19]. Additionally, this segmentation process fails to work effectively in cases where the retinal architecture is altered, e.g. by edema, atrophy or subretinal hemorrhage (Fig. 4b) [19].

**Fig. 5:**
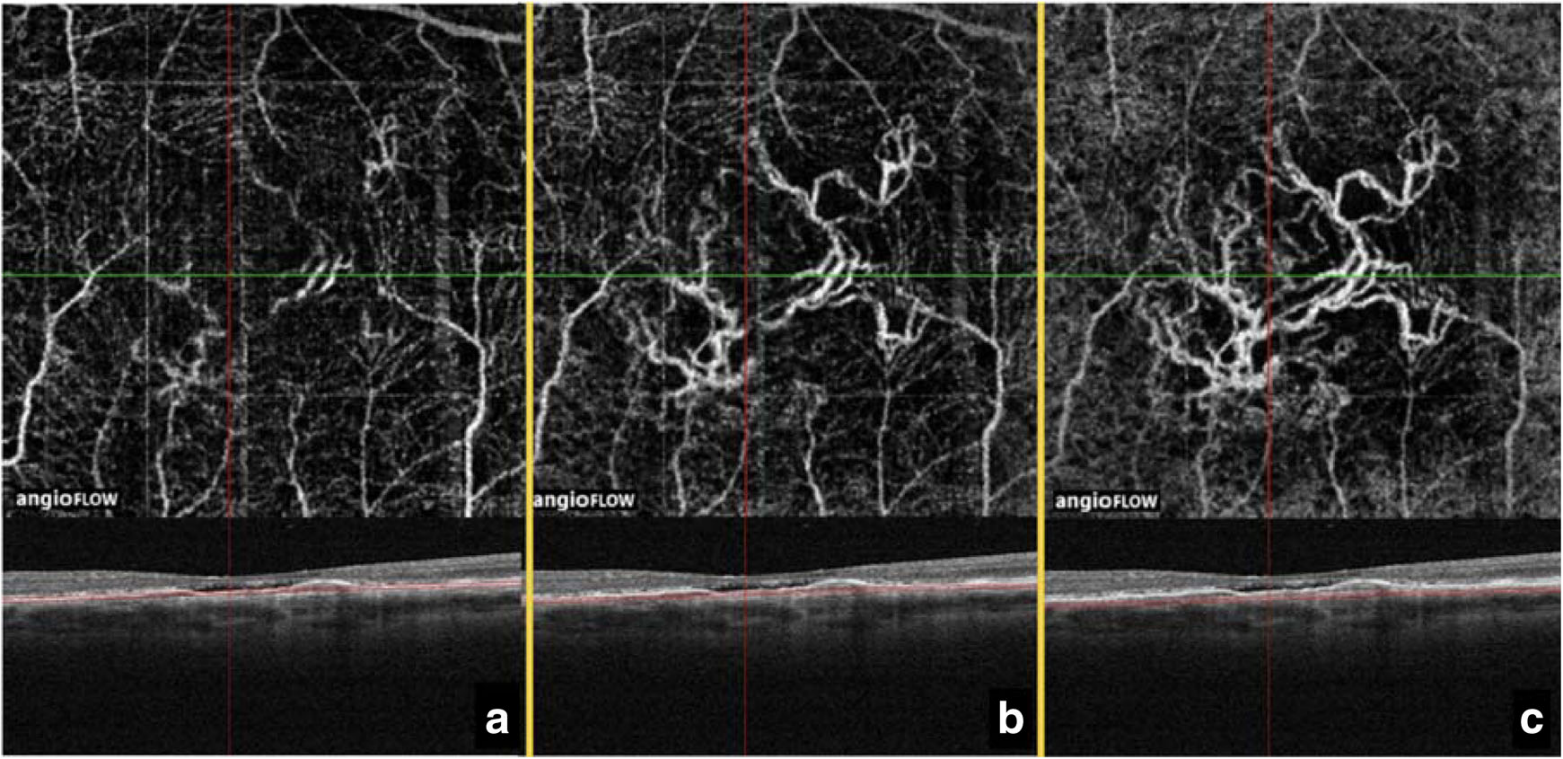
Projection artifacts seen with OCT-A imaging of different tissue layers. **a** At the level of the retinal pigment epithelium, retinal vessels are seen because of projection artifacts. **b** When scanning at the level of the choriocapillaris, the image is still dominated by retinal vasculature projection. **c** Scanning further back into the choroid reveals less projection of retinal vasculature. Please note that the best image of the CNV is obtained using the projection artifact of the same CNV. *CNV* choroidal neovascularization, *OCT-A* optical coherence tomography angiography. Source: Images provided by Giovanni Staurenghi

Projection artifacts are caused by interference from superficial vessels during visualization of deeper tissue structures and are nearly always present in any structure that appears below vasculature (Fig. 5) [19]. As a result, visualization of the corresponding B-scan is required alongside the OCT-A blood flow image for accurate interpretation. Built-in software to correct segmentation error, whereby the OCT-A software corrects for projection artifacts using an automated segmentation process, is under evaluation; however, there is concern that some of the information may be lost, or new sets of image artifacts may be introduced, ultimately leading to misinterpretation by the physician [19].

## Opportunities for OCT-A

Despite the current limitations of OCT-A, it offers a number of potential opportunities to improve the accuracy of diagnosis in clinical practice and the understanding of certain conditions. Accurate identification of the choroidal neovascular membrane within an area of subretinal fluid could provide an insight into the nature of chronic central serous retinopathy, an insufficiently understood condition (thought to be caused by retinal pigment epithelium dysfunction or choroidal hyperpermeability) that leads to fluid accumulation in the subretinal space [21]. The deep-penetrating capabilities of OCT-A offer the opportunity to study differences between deep and superficial blood vessels that cannot be easily distinguished by FA alone.

As a non-invasive technique, OCT-A is suited to cases where dye-based techniques are not appropriate because of patient allergy, or where accurate assessment may be difficult (in which case OCT-A could be used as an additional tool to better interpret the ICGA image). While FA and ICGA are generally considered to be safe techniques, the intravenous dyes used for vascular imaging are contraindicated in pregnancy and may also pose the risk of mild to moderate adverse reactions, e.g. nausea, vomiting, urticaria, and even severe cases of anaphylaxis [2, 22]. The rapid nature of OCT-A image acquisition also makes it ideal for use in cases where the more time-consuming FA and ICGA are not indicated. However, its inability to accurately detect areas of leakage, fluid accumulation, or slow-flowing microaneurysms currently limits its use as the sole diagnostic tool for the majority of macular conditions.

OCT-based imaging biomarkers provide a valuable tool for detecting the early stages of progression of ocular diseases [23]. Moreover, the increasing use of OCT-A in clinical trials and clinical practice may have the potential to identify novel biomarkers for the different retinal diseases [23]. Additionally, in subsets of patients without accumulation of subretinal fluid, OCT-A may provide an important tool for identification of deeper pathologies. There may also be scope for implementing OCT-A imaging for specific pathologies (such as subretinal hyperreflective material lesions) occasionally seen in patients treated for CNV secondary to AMD, providing a potentially useful tool for monitoring disease activity and response to treatment [24].

Implementation of OCT-A technology into a multimodal platform, alongside other established imaging techniques, may provide us with a greater understanding of disease pathways by allowing more accurate assessment of the retinal and choroidal layer microvasculature. For example, Inoue et al. showed that the sensitivity for detecting type 1 neovascularization using combined data from OCT-A and conventional OCT imaging was significantly greater than the sensitivities of either OCT-A or FA imaging alone [13].

## Conclusions

OCT-A is a non-invasive imaging technique with great potential for use in clinical practice. Evidence has demonstrated the effectiveness of OCT-A as an additional tool for retinal disease diagnosis, but its role in disease monitoring remains unclear. With its current practical limitations, considerations must be made regarding how suitable it would be as a primary tool for diagnosis of retinal disease. There is currently no clear consensus on whether OCT-A is useful in all cases or better suited to particular subgroups of patients with retinal disease. At this stage in its development, OCT-A should be considered a complementary diagnostic tool alongside already established imaging modalities, with potential for use in special cases where invasive imaging techniques are inappropriate. While OCT-A imaging is already widely employed in clinical practice, the technology is advancing at a much faster rate than the community’s understanding of and experience with the technique. As advances in OCT-A technology continue to progress, current areas with gaps in knowledge will need to be addressed; the need for standard protocols for OCT-A image acquisition and interpretation, with standardization across all competing OCT-A technologies, has been suggested as the area for primary future focus. Additionally, as OCT-A does not detect leakage, meaning it is not currently possible to differentiate the morphological features of leakage from vascular perfusion, further information is required on the accurate identification of image artifacts to reliably measure signals showing vascular perfusion and to discern between perfusion and leakage.

As a newly developed technology, ongoing research activity should help define best practice for OCT-A in various retinal diseases. Moreover, through integration of qualitative and quantitative OCT-A parameters into clinical trial endpoints, the community-wide understanding of OCT-A technology as a tool for monitoring responses to treatment and guiding treatment decisions could be greatly enhanced.
